# Supplementary material for: Improving musculoskeletal injury surveillance methods in Special Operation Forces: A Delphi consensus study
Source: PLOS Glob Public Health. 2022 Jan 20;2(1):e0000096. doi: 10.1371/journal.pgph.0000096 (PMC10021797; doi:10.1371/journal.pgph.0000096)
Supplement: S1 Table — (DOCX) [file pgph.0000096.s001.docx]

**S1 Table.** A summary of the categories of surveillance information recorded by previous SOF injury epidemiology studies according to the World Health Organisation’s recommended essential data items [1].

| **Article** | **ID** | **Age** | **Sex** | **Intent of injury** | **Place of occurrence** | **Injury type** | **Activity causation** | **MOI** |  |
| --- | --- | --- | --- | --- | --- | --- | --- | --- | --- |
| Pirson & Pirlot (1990) [2] | No | Yes | Yes | No | Yes | No | Yes | Yes |  |
| Linenger et al., (1993) [3] | Yes | Yes | Yes | No | No | Yes | No | No |  |
| Shwayhat et al., (1994) [4] | Yes | Yes | Yes | No | No | Yes | No | No |  |
| Miser & Lillegard (1995) [5] | Yes | Yes | No | No | Yes | Yes | Yes | No |  |
| Kragh et al., (1996) [6] | Yes | Yes | No | No | Yes | Yes | Yes | No |  |
| Ensign et al., (2000) [7] | Yes | Yes | No | No | No | Yes | Yes | No |  |
| Schumacher et al., (2000) [8] | Yes | No | No | No | Yes | Yes | Yes | No |  |
| Kotwal et al., (2004) [9] | Yes | Yes | No | No | Yes | No | Yes | No |  |
| Hughes et al., (2008) [10] | Yes | No | No | No | Yes | Yes | Yes | No |  |
| Lynch & Pallis (2008) [11] | No | No | No | No | No | No | No | No |  |
| Hollingsworth (2009) [12] | Yes | Yes | No | No | No | No | No | No |  |
| Reynolds et al., (2009) [13] | Yes | Yes | No | No | No | Yes | No | No |  |
| Abt et al., (2014) [14] | Yes | Yes | No | No | No | Yes | Yes | Yes |  |
| Teyhen et al., (2015) [15] | Yes | Yes | Yes | No | No | No | No | No |  |
| Lovalekar et al., (2016) [16] | Yes | Yes | Yes | No | No | Yes | Yes | Yes |  |
| Heebner et al., (2017) [17] | Yes | Yes | No | No | No | No | No | No |  |
| Lovalekar et al., (2017) [18] | Yes | Yes | Yes | No | No | Yes | No | No |  |
| Lovalekar et al., (2017) [19] | Yes | No | No | No | No | Yes | Yes | Yes |  |
| Lovalekar et al., (2018) [20] | Yes | Yes | No | No | No | Yes | Yes | Yes |  |
| Teyhen et al., (2018) [21] | Yes | No | No | No | No | No | No | No |  |
| Dijksma et al., (2020) [22] | Yes | Yes | Yes | No | No | No | No | No |  |
| Total studies reporting these variables | 19/21 | 17/21 | 7/21 | 0/21 | 6/21 | 13/21 | 11/21 | 5/21 |  |
| Identification (ID), Mechanism of injury (MOI) | | | | | | | | | |

**References**

1. Holder Y, Peden M, Krug E, Lund J, Gururaj G, Kobusingye O. Injury surveillance guidelines. Geneva: World Health Organisation, 2001.

2. Pirson J, Pirlot M. A study of the influence of body weight and height on military parachute landing injuries. Mil Med. 1990;155(8):383-5.

3. Linenger J, Flinn S, Thomas B, Johnson C. Musculoskeletal and medical morbidity associated with rigorous physical training. Clin J Sport Med. 1993;3(4):229-34.

4. Shwayhat A, Linenger J, Hofherr L, Slymen D, Johnson C. Profiles of exercise history and overuse injuries among United States Navy Sea, Air, and Land (SEAL) recruits. Am J Sports Med. 1994;22(6):835-40. doi: 10.1177/036354659402200616.

5. Miser W, Lillegard W. Injuries and illnesses incurred by an Army Ranger unit during operation Just Cause. Mil Med. 1995;160(8):373-80.

6. Kragh J, Jones B, Amaroso P, Heekin R. Parachuting injuries among Army Rangers: A prospective survey of an elite airborne battalion. Mil Med. 1996;161(7):416-9.

7. Ensign W, Hodgon J, Prusaczyk W, Shapiro D. A survey of self-reported injuries among special boat operators. In: Naval Health Research Centre, editor. San Diego2000.

8. Schumacher J, Creedon J, Pope R. The effectiveness of the parachutist ankle brace in reducing ankle injuries in an airborne ranger battalion. Mil Med. 2000;165(12):944-8.

9. Kotwal R, Meyer D, O’Connor K, Shahbaz B, Johnson T, Sterling Y, et al. Army Ranger casualty, attrition, and surgery rates for airborne operations in Afghanistan and Iraq. Aviat Space Environ Med. 2004;75(10):833-40.

10. Hughes C, Weinrauch P. Military static line parachute injuries in an Australian Commando battalion. ANZ J Surg. 2008;78(10):848-52. Epub 2008/10/31. doi: 10.1111/j.1445-2197.2008.04581.x. PubMed PMID: 18959635.

11. Lynch J, Pallis M. Clinical diagnoses in a Special Forces Group: The musculoskeletal burden. J Spec Oper Med. 2008;8(2):76-80.

12. Hollingsworth D. The prevalence and impact of musculoskeletal injuries during a pre-deployment workup cycle: Survey of a Marine Corps special operations company. J Spec Oper Med. 2009;9:12-5.

13. Reynolds K, Cosio-Lima L, Bovill M, Tharion W, Williams J, Hodges T. A comparison of injuries, limited-duty days, and injury risk factors in infantry, artillery, construction engineers, and special forces soldiers. Mil Med. 2009;174(7):702-8. doi: 10.7205/milmed-d-02-2008.

14. Abt J, Sell T, Lovalekar M, Keenan K, Bozich A, Morgan J, et al. Injury epidemiology of U.S. Army Special Operations Forces. Military Medicine. 2014;179(10):1106-12. Epub 2014/10/01. doi: 10.7205/MILMED-D-14-00078. PubMed PMID: 25269128.

15. Teyhen D, Shaffer S, Butler R, Goffar S, Kiesel K, Rhon D, et al. What risk factors are associated with musculoskeletal injury in US Army Rangers? A prospective prognostic study. Clin Orthop Relat Res. 2015;473(9):2948-58. Epub 2015/05/28. doi: 10.1007/s11999-015-4342-6. PubMed PMID: 26013150; PubMed Central PMCID: PMCPMC4523518.

16. Lovalekar M, Abt J, Sell T, Wood D, Lephart S. Descriptive epidemiology of musculoskeletal injuries in Naval Special Warfare Sea, Air, And Land operators. Mil Med. 2016;181(1):64-9. doi: 10.7205/MILMED-D-14-00655.

17. Heebner NR, Abt JP, Lovalekar M, Beals K, Sell TC, Morgan J, et al. Physical and performance characteristics related to unintentional musculoskeletal injury in special forces operators: A prospective analysis. J Athl Train. 2017;52(12):1153-60. Epub 2017/12/12. doi: 10.4085/1062-6050-52.12.22. PubMed PMID: 29227730; PubMed Central PMCID: PMCPMC5759699.

18. Lovalekar M, Abt J, Sell T, Lephart S, Pletcher E, Beals K. Accuracy of recall of musculoskeletal injuries in elite military personnel: A cross-sectional study. BMJ open. 2017;7(12). doi: 10.1136/bmjopen-2017-017434. PubMed PMID: 29247087; PubMed Central PMCID: PMCPMC5736038.

19. Lovalekar M, Perlsweig K, Keenan K, Baldwin T, Caviston M, McCarthy A, et al. Epidemiology of musculoskeletal injuries sustained by Naval Special Forces Operators and students. J Sci Med Sport. 2017;20:51-6. doi: 10.1016/j.jsams.2017.09.003.

20. Lovalekar M, Johnson C, Eagle S, Wohleber M, Keenan K, Beals K, et al. Epidemiology of musculoskeletal injuries among US Air Force special tactics operators: An economic cost perspective. BMJ Open SEM. 2018;4(1). doi: 10.1136/bmjsem-2018-000471.

21. Teyhen D, Goffar S, Shaffer S, Kiesel K, Butler R, Tedaldi A, et al. Incidence of musculoskeletal injury in US Army unit types: A prospective cohort study. J Orthop Sports Phys Ther. 2018;48(10):749-57. Epub 2018/05/23. doi: 10.2519/jospt.2018.7979. PubMed PMID: 29787695.

22. Dijksma I, Zimmermann W, Hertenberg E, Lucas C, Stuiver M. One out of four recruits drops out from elite military training due to musculoskeletal injuries in the Netherlands Armed Forces. BMJ Mil Health. 2020:1-5. Epub 2020/03/07. doi: 10.1136/bmjmilitary-2020-001420. PubMed PMID: 32139408.
