# Supplementary material for: Improving musculoskeletal injury surveillance methods in Special Operation Forces: A Delphi consensus study
Source: PLOS Glob Public Health. 2022 Jan 20;2(1):e0000096. doi: 10.1371/journal.pgph.0000096 (PMC10021797; doi:10.1371/journal.pgph.0000096)
Supplement: Attachment — Submitted filename: Response to Reviewers.docx [file pgph.0000096.s002.docx]

| Feedback received | Author’s response |
| --- | --- |
| Editor Comments | |
| 1. Please provide a complete Data Availability Statement in the submission form, ensuring you include all necessary access information or a reason for why you are unable to make your data freely accessible. Note that it is not acceptable for the authors to be the sole named individuals responsible for ensuring data access. | Data availability statement has been changed to “All data are in the manuscript and/or supporting information files" |
| 2. Please amend your detailed Financial Disclosure statement | Changed |
| 3. Please review your reference list to ensure that it is complete and correct | Reference style has been changed to Vancouver.  Journals now include DOI.  No retracted papers have been cited. |
| 4. Elaborate further specifically on the global public health implications of these findings. | This has been added to first paragraph of the discussion section, line 194 and now reads  “This study obtained expert opinion on the need to develop SOF injury surveillance guidelines and new knowledge in working towards improving injury surveillance methods in the military and SOF globally. There was strong agreement in support of consistent approaches across nations. Consistent methods between nations will allow the sharing of knowledge and assist in joint efforts towards addressing injury mitigation in military organisations globally. While it is appreciated that a completely standardised approach is not always realistic, with a pragmatic view, we encourage those conducting surveillance investigations to follow the guidelines presented below where possible. These guidelines provide a step forward in supporting accurate, consistent and reliable injury surveillance in SOF. It is anticipated that these guidelines will require periodic evaluation in response to changes in surveillance needs and capabilities. “ |
| Reviewer 1 | |
| 5. Include discussion on secrecy of SOF operations as a potential barrier to data collection | This has been acknowledged, paragraph starting on line 347.  “The classified nature of SOF operations was infrequently raised as a barrier to data collection. Lack of discussion on this potential limitation could be due to the questionnaire being designed in a non-sensitive manner, and thus when presented to participants, concerns were not raised. Data items where information sensitivity was briefly highlighted as a potential issue related to the place of occurrence and activity causation. One expert’s comment suggested that as historical information indicates most injuries occur from physical training, a non-sensitive activity, security requirements are not a significant barrier for effective injury surveillance. Surveillance should always be conducted in alignment with organisational security requirements.” |
| 6. Table 3 there is a typo on item 13. It should read n = 16, not n = 100. | This has been corrected. |
| Reviewer 2 | |
| 7. Further explanation to what an agreed upon consensus could provide for injury surveillance | This has been explained further in the introduction, line 75 and also in the first paragraph of the discussion section, line 197. |
| 8. Is this sample size of interviews considered acceptable among other Delphi studies? | Yes, this has been acknowledged and explained within the limitations and strengths section, line 377 and reads  “There are no statistically bound sample size criteria for Delphi studies. Our sample size is consistent with participant numbers in other Delphi studies (18). Previous research has indicated that reliable outcomes can be obtained from such sizes when using experts with similar training and knowledge in a given topic with a limited number of experts in a respective field (50). Given the demographical characteristics of our sample, we believe our sample size was sufficient to achieve reliable results for the purpose of this study.”  . |
| 9. Can the authors please make a justification why they believe this distribution of size of respondents is sufficient for their analysis? | Yes, please refer to comment 8. |
| 10. More discussion to the limitations of this study design would be helpful but include how this study design and findings are critical for future studies. | We have addressed this by adding to the discussion the strengths of the Delphi study design (line 357) and elaborating on the limitations and discussion on sample size (line 377). |
| 11. Additional discussion to provide what / how you feel these findings need to be translated to further research will be helpful to convey the progression of this work. | Additional discussion surrounding recommendations to future research has been made related to investigating injury underreporting (line 340) and developing injury causation codes (line 370). |
